# Supplementary material for: Identification of Serum Oxylipins Associated with the Development of Coronary Artery Disease: A Nested Case-Control Study
Source: Metabolites. 2022 May 30;12(6):495. doi: 10.3390/metabo12060495 (PMC9231201; doi:10.3390/metabo12060495)
Supplement: Supplementary file 1 [file metabolites-12-00495-s001.zip › metabolites-1674203-supplementary-fixed.pdf]

Table S1. Odd ratio (OR) and 95% confidence interval (CI) for CAD associated with tertile of each oxylipin.

Oxylipins	Borderline (ppm)	Number of cases/controls	Crude Model OR (95% CI)	Model A OR (95% CI)
**13-oxo-ODE**				
Low tertile	<0.0055	18/52	Ref.	Ref.
Middle tertile	0.0055-0.006	31/40	2.23 (1.11, 4.95)*	2.90 (1.29, 6.52)*
High tertile	≥0.006	28/42	1.88 (0.91, 3.86)	2.01 (0.95, 4.28)
**5-HETE**				
Low tertile	<0.0173	30/40	Ref.	Ref.
Middle tertile	0.0173-0.0180	20/51	0.54 (0.26, 1.12)	0.48 (0.22, 1.06)
High tertile	≥0.0180	27/43	0.80 (0.39, 1.65)	0.76 (0.36, 1.61)
**PGD2/PGE2**				
Low tertile	<0.001001	37/33	Ref.	Ref.
Middle tertile	0.001001-0.001005	20/51	0.36 (0.17, 0.73)*	0.35 (0.17, 0.73)*
High tertile	≥0.001005	20/50	0.39 (0.20, 0.78)*	0.37 (0.18, 0.76)*
**15-deoxy-PGJ2**				
Low tertile	<0.001002	29/41	Ref.	Ref.
Middle tertile	0.001002-0.00101	30/41	1.03 (0.52, 2.07)	1.08 (0.53, 2.18)
High tertile	≥0.00101	18/52	0.43 (0.20, 0.96)*	0.38 (0.17, 0.86)*

Model A: Adjusted for drinking, smoking, education and experimental batch.

* *p*-value < 0.05, ***p*-value < 0.01. Ref. = reference group.

Table S2. ROC results.

Prediction Model	ROC association test				Contrast test
	AUC	SE	95% CI		p-value
**Basic**	0.627	0.04	0.55	0.71	**ref.**
**Basic + 13-oxoODE**	0.659	0.04	0.58	0.73	0.190
**Basic + 5-HETE**	0.650	0.04	0.57	0.72	0.310
**Basic + 15-deoxyPGJ2**	0.675	0.04	0.60	0.75	0.110
**Basic + PGD2**	0.712	0.04	0.64	0.78	***0.015****
**Basic + PGD2+15-deoxyPGJ2**	0.740	0.04	0.67	0.81	***0.004****
**Basic + PGD2+15-deoxyPGJ215+13-oxoODE**	0.755	0.03	0.69	0.82	***0.0012****
**Basic + PGD2+15-deoxyPGJ2+13-oxoODE+5-HETE**	0.762	0.03	0.70	0.83	***0.001****

Basic model: age, sex, and status of hypertension, diabetes, hypertriglyceridemia, hypercholesterolemia, smoking, drinking, and education. ROC: Receiver operating characteristic. AUC: Area under the curve. SE: Standard Error. CI: confidence interval.

Table S3. The information of 46 analyzed oxylipins

No.	Oxylipin Name	Abbreviation	Parent ion (m/z)	Product ion (m/z)	Retention Time (min)
1	9-hydroxyoctadecadienoic acid	9-HODE	295.2	171±0.25	10.98
2	13-hydroxyoctadecadienoic acid	13-HODE	295.2	195±0.25	10.74
3	9-oxooctadecadienoic acid	9-oxo-ODE	293.2	185.1±0.25	10.69
4	13-oxooctadecadienoic acid	13-oxo-ODE	293.2	113±0.25	10.69
5	9,10-dihydroxyoctadecenoic acid	9,10-DiHOME	313.2	201.2±0.25	9.6
6	12,13-dihydroxyoctadecenoic acid	12,13-DiHOME	313.2	183.2±0.25	9.45
7	9,10,13-trihydroxyoctadecenoic acid	9,10,13-triHOME	329.2	171.1±0.25	8.11
8	9,12,13-trihydroxyoctadecenoic acid	9,12,13-triHOME	329.2	211.1±0.25	8.11
9	9,10-epoxyoctadecenoic acid	9,10-EpOMe	295.2	171.1±0.25	10.99
10	12,13-epoxyoctadecenoic acid	12,13-EpOMe	295.2	195.2±0.25	10.74
11	5-Hydroxyeicosatetraenoic acid	5-HETE	319.2	115.1±0.25	11.67
12	8-Hydroxyeicosatetraenoic acid	8-HETE	319.2	301.2±0.25	11.28
13	9-Hydroxyeicosatetraenoic acid	9-HETE	319.2	123.1±0.25	11.28
14	11-Hydroxyeicosatetraenoic acid	11-HETE	319.2	167.2±0.25	11.1
15	12-Hydroxyeicosatetraenoic acid	12-HETE	319.2	179.2±0.25	11.23
16	15-Hydroxyeicosatetraenoic acid	15-HETE	319.2	301.4±0.25	11.23
17	19-Hydroxyeicosatetraenoic acid	19-HETE	319.2	275.1±0.25	11.24
18	20-Hydroxyeicosatetraenoic acid	20-HETE	319.2	275.2±0.25	11.24
19	5-oxoeicosatetraenoic acid	5-oxo-ETE	317.2	273.2±0.25	10.92
20	15-oxoeicosatetraenoic acid	15-oxo-ETE	317.2	113.1±0.25	10.31
21	Leukotriene A4	LTA4	311	183±0.25	12.1
22	Leukotriene B4	LTB4	335.2	195.1±0.25	9.94
23	Lipoxin A4	LXA4	351.1	115±0.25	8.21
24	Lipoxin B4	LXB4	351	217±0.25	7.08
25	Prostaglandin E2/D2	PGE2/PGD2	351.2	271.3±0.25	7.07
26	Prostaglandin B2/J2	PGB2/PGJ2	333.2	235.3±0.25	8.4
27	6-keto-prostaglandin F1α	6-keto-PGF1α	383.4	187±0.25	7.45
28	15-deoxy-prostaglandinJ2	15-deoxy-PGJ2	315.2	271.3±0.25	9.86
29	Prostaglandin F2α	PGF2α	353.2	309.3±0.25	7.51
30	Thromboxane B2	TXB2	369.2	169.1±0.25	7.13
31	5,6-epoxyeicosatrienoic acid	5,6-EET	319.2	191±0.25	11.25
32	8,9-epoxyeicosatrienoic acid	8,9-EET	319.2	123±0.25	11.21
33	11,12-epoxyeicosatrienoic acid	11,12-EET	319.2	167±0.25	11.16
34	14,15-epoxyeicosatrienoic acid	14,15-EET	319.2	219.3±0.25	11
35	5,6-dihydroxyeicosatrienoic acid	5,6-DHET	337.2	145.1±0.25	10.14

36	8,9-dihydroxyeicosatrienoic acid	8,9-DHET	337.2	127.1±0.25	9.9
37	11,12-dihydroxyeicosatrienoic acid	11,12-DHET	337.2	167.1±0.25	10.13
38	14,15-dihydroxyeicosatrienoic acid	14,15-DHET	337.2	207.1±0.25	9.89
39	11,12,15-Trihydroxyeicosatrienoic acid/Tetrahydrofurandiols	11,12,15-THET /THFdiols	353.2	167.1±0.25	8.97
40	Docosahexaenic Acid	DHA	327.1	283±0.25	12.99
41	Maresin	Maresin	359.3	250±0.25	16.27
42	17-hydroxydocosahexaenoic acid	17-HDoHE	343.2	281±0.25	11.33
43	10,17-Dihydroxydocosahexaenoic acid	10,17-DiHDoHE	359	206±0.25	9.47
44	Eicosapentaenoic acid	EPA	301.1	257±0.25	12.31
45	15-hydroxyeicosapentaenoic acid	15-HEPE	317	219±0.25	16.4
46	Resolvin D1	Resolvin D1	375.3	121±0.25	8.26

Table S4. Concentrations of the Oxylipins (ppm)

Metabolites	Control (N=134)		Case (N=77)	
	Mean	SD	Mean	SD
**9-HODE**	0.0294	0.037	0.0301	0.0376
**13-HODE**	0.0599	0.028	0.0625	0.0345
**9-oxo-ODE**	0.0058	0.0007	0.0060	0.0011
**13-oxo-ODE**	0.0058	0.0017	0.0061	0.0013
**9,10-DiHOME**	0.0203	0.011	0.0219	0.013
**12,13-DiHOME**	0.0122	0.009	0.0107	0.0079
**9,10,13-triHOME**	0.0685	0.125	0.0761	0.143
**9,12,13-triHOME**	0.0470	0.084	0.0521	0.098
**9,10-EpOMe**	0.0352	0.040	0.0377	0.038
**12,13-EpOMe**	0.0585	0.025	0.0614	0.031
**5-HETE**	0.0182	0.002	0.0181	0.0013
**8-HETE**	0.0243	0.0085	0.0246	0.0049
**9-HETE**	0.0167	0.0004	0.0166	0.00018
**11-HETE**	0.0183	0.003	0.0182	0.0017
**12-HETE**	0.0176	0.002	0.0176	0.0015
**15-HETE**	0.0260	0.013	0.0252	0.0061
**19-HETE**	0.0296	0.049	0.0254	0.0059
**20-HETE**	0.0253	0.041	0.0215	0.0022
**5-oxo-ETE**	0.0190	0.007	0.0181	0.0020
**15-oxo-ETE**	0.0168	0.001	0.0166	0.00014
**LTA4**	5.106	3.901	4.824	2.951
**LTB4**	0.0167	0.0012	0.0166	0.00026
**LXA4**	0.0165	0.00003	0.0165	0.00002
**LXB4**	0.0170	0.0003	0.0169	0.00028
**5,6-EET**	0.0907	0.015	0.0889	0.008
**8,9-EET**	0.0962	0.065	0.0879	0.009
**11,12-EET**	0.316	0.424	0.298	0.207
**14,15-EET**	0.135	0.217	0.119	0.032
**5,6-DHET**	0.095	0.064	0.089	0.015
**8,9-DHET**	0.085	0.013	0.084	0.006
**11,12-DHET**	0.103	0.021	0.102	0.020
**14,15-DHET**	0.131	0.032	0.132	0.036
**Diols**	0.083	0.00091	0.083	0.0015
**PGF2a**	0.00102	0.00002	0.00101	0.00002
**TXB2**	0.00102	0.00001	0.00102	0.00001

**15-deoxy-PGJ2**	0.00107	0.00014	0.00106	0.00008
**PGE2/PGD2**	0.001015	0.000047	0.001013	0.000066
**PGJ2**	0.001009	0.000011	0.001008	0.000008
**DHA**	12.226	7.579	11.324	5.061
**17-HDoHE**	0.607	0.534	0.722	0.762
**EPA**	0.747	1.689	0.537	0.405
**15-HEPE**	1.507	1.421	1.452	0.961

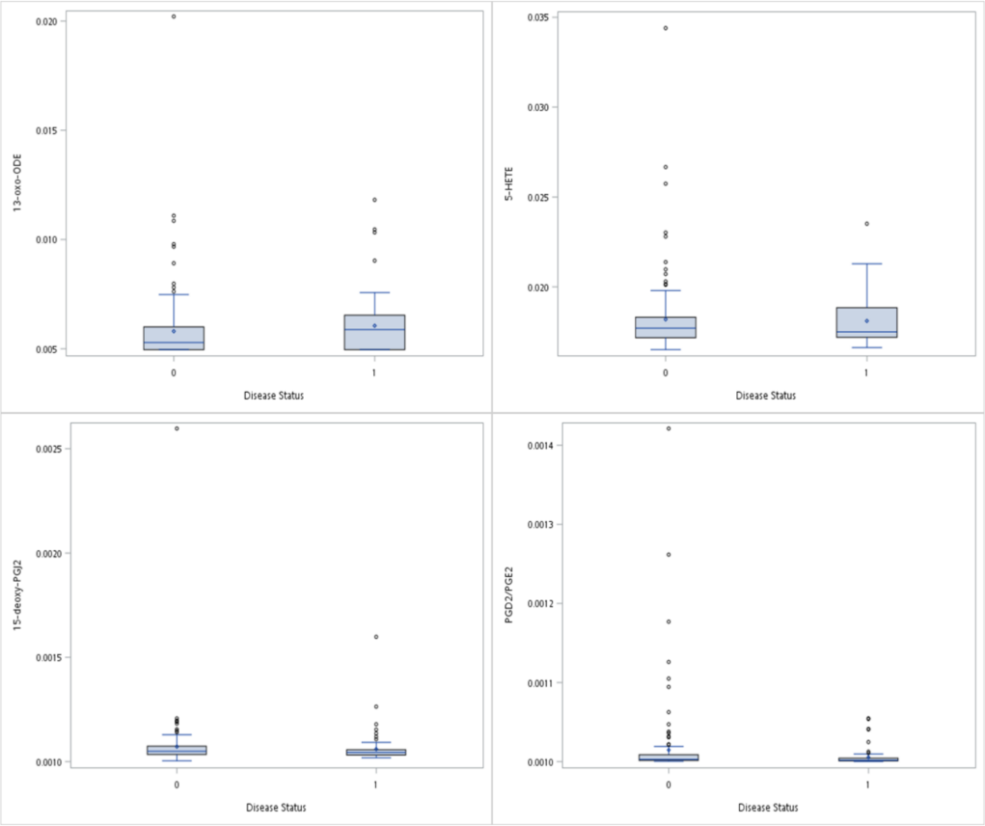

Figure S1. Boxplot of the four identified oxylipins (ppm). (0: control, 1: case)

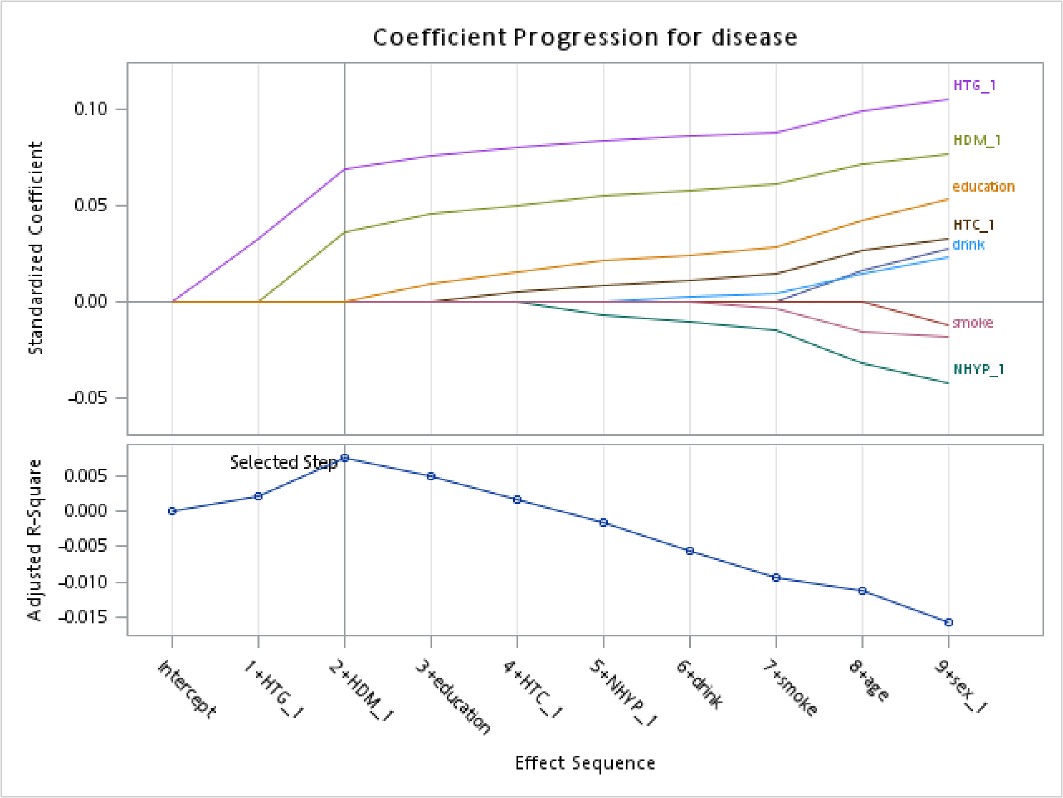

Figure S2. LASSO selected step: NHYP_1: hypertension, HDM_1: diabetes mellitus, HTG_1: hypertriglyceridemia, HTC_1: hypercholesterolemia.

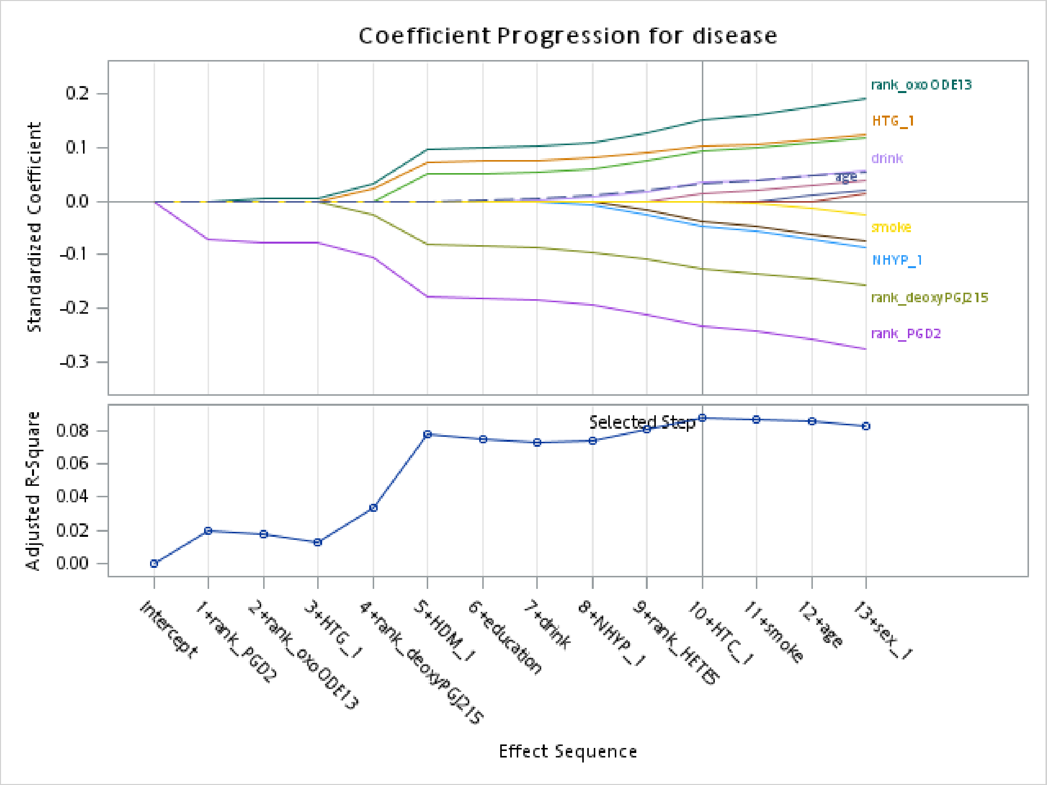

Figure S3. LASSO selected step: NHYP_1: hypertension, HDM_1: diabetes mellitus, HTG_1: hypertriglyceridemia, HTC_1: hypercholesterolemia.

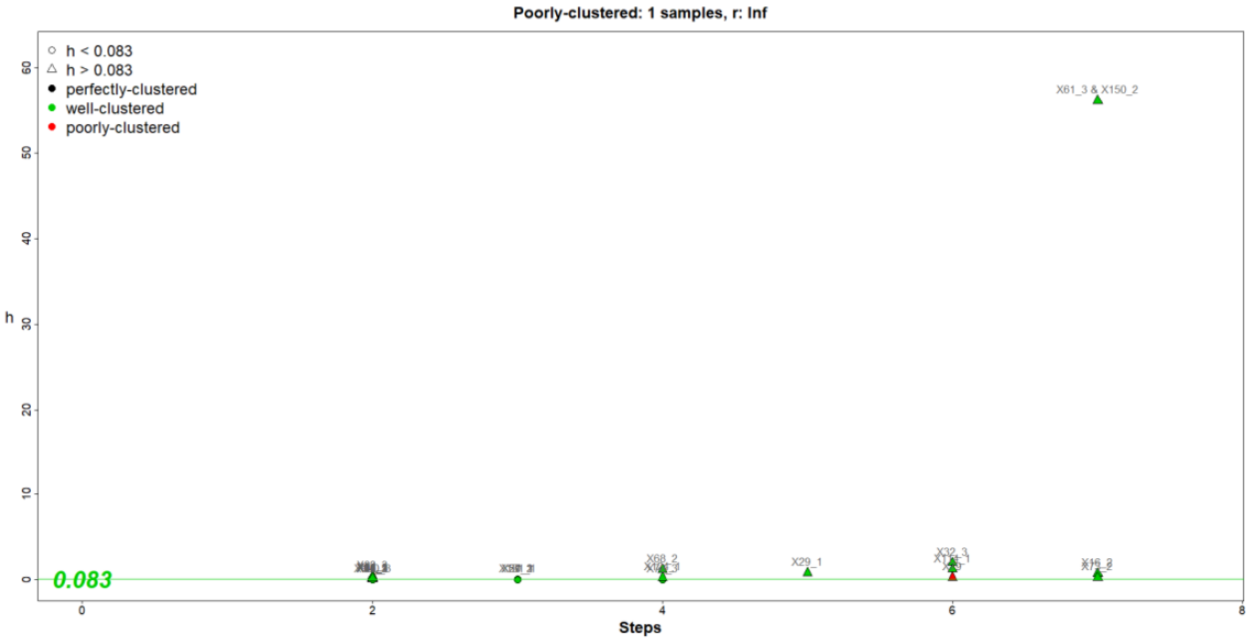

Figure S4. Quality control plot for sample filtering. In this plot, the vertical axis indicates the log-transformed distance h, and the horizontal axis indicates the number of steps at which all replicate samples of a subject were joined together. Each symbol indicates a subject. All subjects

were classified as perfectly clustered (black), well clustered (green), or poorly clustered (red). The reference line (s-value = 0.083) was obtained using the normality-based method based on all peaks. Subjects with a log-transformed distance $h \leqq 0.083$ are indicated by a circle, whereas the other subjects are indicated by a triangle.
